# What supports do health system organizations have in place to facilitate evidence-informed decision-making? a qualitative study

**DOI:** 10.1186/1748-5908-8-84

**Published:** 2013-08-06

**Authors:** Moriah E Ellen, Gregory Léon, Gisèle Bouchard, John N Lavis, Mathieu Ouimet, Jeremy M Grimshaw

**Affiliations:** 1 Centre for Health Economics and Policy Analysis, McMaster University, 1280 Main Street West, CRL 209, Hamilton, ON, Canada L8S 4K1; 2 Department of Clinical Epidemiology and Biostatistics, McMaster University, 1280 Main Street West, CRL 209, Hamilton, ON, Canada L8S 4K1; 3 Jerusalem College of Technology, Hava’ad Haleumi 21, Jerusalem, Israel 93721; 4 Israeli Center for Technology Assessment in Health Care, Tel Hashomer 52621, Israel; 5 Department of Political Science, Université Laval, Pavillon Charles-De Koninck, 1030 avenue des Sciences humaines, office 4453, Quebec, QC G1V 0A6, Canada; 6 McMaster Health Forum, McMaster University, 1280 Main Street West, MML-417, Hamilton, ON, Canada L8S 4L6; 7 Department of Political Science, McMaster University, 1280 Main Street West, CRL 209, Hamilton, ON, Canada L8S 4K1; 8 Department of Global Health and Population, Harvard School of Public Health, 677 Huntington Ave, Boston, MA 02115-6018, USA; 9 Research Axis of Public health and practice-changing research, Centre Hospitalier Universitaire de Québec Research Centre, Hôpital Saint-François d’Assise, 10 rue de l’Espinay, office D6-726, Quebec, QC G1L 3L5, Canada; 10 Clinical Epidemiology Program, Ottawa Hospital Research Institute, Ottawa Hospital - General Campus, Centre for Practice-Changing Research (CPCR), 501 Smyth Road, Room 1286, Ottawa, Canada K1H 8L6; 11 Department of Medicine, University of Ottawa, Ottawa Hospital - General Campus, Centre for Practice-Changing Research (CPCR), 501 Smyth Road, Room 1286, Ottawa, Canada K1H 8L6

## Abstract

**Background:**

Decisions regarding health systems are sometimes made without the input of timely and reliable evidence, leading to less than optimal health outcomes. Healthcare organizations can implement tools and infrastructures to support the use of research evidence to inform decision-making.

**Objectives:**

The purpose of this study was to profile the supports and instruments (*i.e*., programs, interventions, instruments or tools) that healthcare organizations currently have in place and which ones were perceived to facilitate evidence-informed decision-making.

**Methods:**

In-depth semi-structured telephone interviews were conducted with individuals in three different types of positions (*i.e*., a senior management team member, a library manager, and a ‘knowledge broker’) in three types of healthcare organizations (*i.e*., regional health authorities, hospitals and primary care practices) in two Canadian provinces (*i.e*., Ontario and Quebec). The interviews were taped, transcribed, and then analyzed thematically using NVivo 9 qualitative data analysis software.

**Results:**

A total of 57 interviews were conducted in 25 organizations in Ontario and Quebec. The main findings suggest that, for the healthcare organizations that participated in this study, the following supports facilitate evidence-informed decision-making: facilitating roles that actively promote research use within the organization; establishing ties to researchers and opinion leaders outside the organization; a technical infrastructure that provides access to research evidence, such as databases; and provision and participation in training programs to enhance staff’s capacity building.

**Conclusions:**

This study identified the need for having a receptive climate, which laid the foundation for the implementation of other tangible initiatives and supported the use of research in decision-making. This study adds to the literature on organizational efforts that can increase the use of research evidence in decision-making. Some of the identified supports may increase the use of research evidence by decision-makers, which may then lead to more informed decisions, and hopefully to a strengthened health system and improved health.

## Background

Ensuring the use of research evidence in health system management, policy- and decision-making is an important challenge [1]. Significant worldwide investments that are made in biomedical and health research are underutilized because of challenges in knowledge translation. Evidence shows that health systems frequently fail to optimally use research evidence, which leads to inefficiencies, reduced quantity and quality of life for citizens, and lost productivity [2]. Health systems research evidence is not always communicated effectively or in a timely manner, and health system managers, policy- and decision-makers do not always have the skills, tools and capacity to find and use evidence [3,4]. Knowledge translation (KT) has emerged as a paradigm to address many of these challenges and start closing the ‘know-do’ gap [1]. KT is defined as “a dynamic and iterative process that includes synthesis, dissemination, exchange and ethically-sound application of knowledge to improve the health of Canadians, provide more effective health services and products and strengthen the healthcare system” [5].

Numerous factors can determine and influence the use of research in management and decision-making, such as the knowledge-users’ skills in finding and applying the evidence or appropriate access to research evidence. Managing current knowledge is a starting point, but is probably not enough to ensure the effective use of research evidence to inform decision-making given that there are challenges that operate at multiple levels, such as the health system (*e.g.*, financial disincentives), organization (*e.g.*, lack of appropriate equipment), teams (*e.g.*, local standards not in line with desired practice), professionals (*e.g.*, knowledge and skills), and patients (*e.g.*, poor adherence to medical advice) [6]. Barriers to accessing evidence, such as poor technical infrastructures and inadequate journal subscriptions, as well as cognitive barriers such as the lack of knowledge regarding how to find, appraise, and apply the appropriate evidence, are challenges to KT for health system managers, decision-makers, and policy-makers [7-9]. Numerous challenges are often present at different levels of the healthcare system, and as a result, KT approaches and activities need to address the various levels of dynamics that come into play within healthcare organizations.

Multiple factors can be associated with the use of research evidence by decision-makers, including the timeliness and relevance of the research evidence, personal contact with researchers, and inclusion of summaries with actionable messages [10,11]. These findings have led to the development of KT approaches and tools that target policy-makers and senior health system managers to increase their use of research evidence in decision-making [12,13]. The three main KT approaches to target these groups that have been discussed in the literature are ‘push,’ ‘pull,’ and ‘exchange’ efforts [4,14]. ‘Push’ efforts include activities usually undertaken by researchers or intermediary groups (either intermediary organizations or intermediary in the process, *i.e*., a position that is in between research producers and users such as librarians or knowledge brokers) to appropriately package and disseminate research evidence to potential knowledge-users. ‘Pull’ efforts focus on the efforts by health system managers and policy-makers to access and use research evidence. ‘Exchange’ activities focus on building and maintaining relationships between researchers and health system managers and policy-makers.

The purpose of the present study was to profile the supports that healthcare organizations (*i.e*., regional health authorities, hospitals, and primary care practices) in two Canadian provinces (*i.e*., Ontario and Quebec) currently have in place to facilitate evidence-informed decision-making (EIDM), based on push, pull and exchange efforts. We defined these supports as any instrument or intervention (*i.e.*, positions, infrastructures, programs, tools or devices) implemented in healthcare organizations or broader health systems of which they are a part in order to facilitate access, dissemination, exchange, and/or use of research evidence [14]. Based on a recent environmental scan and scoping review, we were unable to identify any studies evaluating the effects of a full research knowledge infrastructure on the use of evidence by health system managers and policy-makers. Thus, we developed a framework that addresses the infrastructure components that an organization or health system can have in place to facilitate the use of research evidence in management and decision-making (for further details, please see Ellen and colleagues, 2011 [14]).

The present qualitative research is part of a broader study to document organizational commitments promoting access to and use of research evidence by managers and policy-makers in various Canadian healthcare organizations. The objective was to understand the current mix of supports these organizations have in place and their views about the most important supports in facilitating the use of research evidence to inform decision-making.

## Methods

In-depth, semi-structured telephone interviews were conducted in two Canadian provinces (Ontario and Quebec) in three types of health system organizations: regional health authorities (RHAs), hospitals, and primary care practices (PCPs). Semi-structured interviews were preferred over other study designs because they allow participants to respond freely, to focus on the area that they perceive as most influential to their organization, and permit the interviewer to probe issues that may be of interest but that are not specifically addressed by the interview guide [15,16]. This approach is also recognized as an effective research method in fields where little research data are available [17]. As limited data is presently available in the field of KT with respect to the organizational infrastructures needed to support evidence-informed decisions, this approach was deemed the most efficient method.

### Developing the interview guide

The interview guide was based on an environmental scan and scoping review that identified the potential supports that an organization can have in place to facilitate the use of evidence in management and decision-making [14]. The seven main domains of supports identified in the scoping review were: climate for research use, research production, push efforts to link research to action, facilitating pull efforts to link research to action, pull efforts to link research to action, linkage and exchange efforts, and evaluation efforts [4,14]. The focus of the interviews was to explore the current supports these organizations have in place and, of those, which are perceived to have added value to the participant’s organization (and which have not). We told participants that when we refer to evidence, we include academic research outputs (*i.e.*, articles, research reports, and books) and population and health system data (*i.e.*, surveillance data and service utilization data).

### Selecting and recruiting the sample

The sample was drawn at three levels: the provincial, the organizational, and the employee level. This three-stage sampling process enabled us to identify the individuals in the organizations and provinces that have implemented a wide range of interventions to support the use of research evidence in decision-making.

First, we purposely chose to conduct the interviews in Ontario and Quebec, since these are two of the largest provinces in Canada that account for more than 50% of the Canadian population. The two provinces have already heavily invested in knowledge translation initiatives. Our goal was to learn from ‘high-performers,’ and to understand what they have implemented, and what they view as the most important elements, so that others can learn from these initiatives.

Second, we purposely sampled at the organizational level. We selected three different types of health system organizations (RHAs, hospitals and PCPs) because these organizations are accountable for the funding and/or the delivery of the bulk of healthcare services in Ontario and Quebec. We purposely sampled organizations that have already participated in strategic behavior with respect to KT activities. We did this by examining the publicly available list of participants and organizations that have been a part of the Canadian Health Services Research Foundation’s Executive Training for Research Application (EXTRA) program. The EXTRA program aims to develop skills and leadership related to the use of health systems evidence in Canadian health system managers and policy-makers (for further details, please see the study protocol) [14]. We chose three RHAs, five to six hospitals, and six PCPs in each province.

Third, we purposely sampled position types. Within each organization, we strived to interview individuals in three different types of positions that could provide us with an overarching view, as well as different perspectives, on the use of research evidence in decision-making. We targeted the following: a senior management team member (who was more focused on organizational infrastructure), a library or resource centre manager (who was more focused on technical infrastructure), and a knowledge broker (or someone in a position that implies supporting evidence-informed decision-making in the organization, management and delivery of health services). Once potential participants were contacted, we also used the chain-referral sampling technique to identify individuals within the organization who, in their view, were best suited to answer our research questions [18].

We recognized that not all health system organizations would have all three types of positions; therefore, depending on the organization’s size and type, we interviewed between one and three participants in each. We attempted to conduct at most 18 interviews in RHAs (9 each in Ontario and Quebec), 30 in hospitals (15 in Ontario and 15 in Quebec), and 24 in PCPs (12 each in Ontario and Quebec), for a total potential of 72 interviews. A cover letter, project summary, and consent form were sent to each potential participant. Follow-up emails and phone calls were made, when necessary.

### Data analysis

Interviews were audio-recorded, transcribed, deidentified, checked for accuracy, and then analyzed thematically. NVivo 9, a qualitative data analysis software (QSR International, Cambridge, MA) was used for data management and coding. Field notes were kept during the interviews and were referred to during the data analysis. A constant comparative method was used for the thematic analysis of the interview data. First and second level nodes were developed based on our KT framework [14]. Third level nodes were developed inductively by ME, GB and GL throughout the coding and analysis, and were based on themes that were recurrent in the interviews. Interviews were analyzed in clusters. First, we read the entire interview to get a sense of the whole interview and initial impressions. Second, we coded units of text into nodes and subnodes in a second read, and compared initial codes. Three researchers, (ME, GB and GL) conducted the initial coding and comparison. We ensured that at least two researchers coded each interview, and, together with JL and MO, we revisited the overall coding framework and revised it until agreement was reached. Third, we analyzed the data for the KT elements that the participants said they currently have in place, and the KT elements that the participants considered as their top three to support evidence-informed decision-making.

For each coded element, we calculated the frequencies and relative percentages of total number of participants by organization, province, and position type. Only KT elements that were coded for ≥ 20% of the total number of participants were deemed of sufficient frequency for inclusion. Next, we searched for differences in coded responses that were ≥ 50% of the total number of participants within organization and position type. We utilized the following frequency taxonomy to describe the results: ‘all’ refers to 100%, ‘most’ refers to 67% to 99%, ‘many’ refers to 33% to 66%, ‘some’ refers to 1% to 32%, and ‘none’ refers to 0% of total participants. An effort was made in the results section to include only elements and sub-elements that were mentioned by all or most (*i.e*., more than 66%) of the participants. However, in some domains (*i.e*., research production and push efforts), none of the sub-elements were mentioned by more than 66% of the participants, and therefore sub-elements that were mentioned by 33% to 66% of the participants were included. Frequency of responses is noted selectively to illustrate divergence or differences in endorsement of identified constructs. Where differences are noted, these are qualitative in nature and were not tested for statistical significance.

### Ethics

The study protocol was submitted to and approved by the Hamilton Health Science and the CHUQ Research Ethics Boards, for Ontario and Quebec, respectively.

## Results

We sent out 104 invitations by email with the goal of having a total of 72 respondents; 27 did not respond, and 20 declined our invitation. Some non-response was due to turnover, and some or potential respondents noted that they were not the correct individual to participate in the interview. Thus, 57 interviews were conducted in 25 organizations (*i.e*., 3 RHAs, 5 to 6 hospitals and 2 to 4 PCPs) in Ontario and Quebec. Library managers or knowledge brokers were not present in all selected institutions and, therefore, 58% of the interviews were conducted with senior managers (see Table 1 for a breakdown of interview participants by province, organization and position type). Furthermore, it was challenging to identify and recruit the appropriate participants in PCPs for three possible reasons; limited information available on their websites (if any), internal policies that limit or restrict research projects (notably in Quebec), and the positions we were targeting did not exist. Therefore, only 9 interviews (*i.e*., 16%) were conducted in PCPs.

**Table 1:** Interview participants by organization and position type

**Province**	**Number of interview participants (% of total)**						
	**Total**	**Organization**			**Position**		
		**PCP**^**1 **^**(7 organizations)**	**Hospital (11 organizations)**	**RHA**^**2 **^**(6 organizations)**	**Senior manager**	**Knowledge broker**	**Library manager**
Ontario	29 (51%)	3 (5%)	14 (25%)	8 (14%)	19 (33%)	5 (9%)	5 (9%)
Quebec	28 (49%)	6 (11%)	18 (32%)	8 (14%)	14 (25%)	6 (11%)	8 (14%)
Total	57 (100%)	9 (16%)	32 (56%)	16 (28%)	33 (58%)	11 (20%)	13 (23%)

^1^*PCP* Primary Care Practices.

^2^*RHA* Regional Health Authority.

Coding of the seven main domains developed in our framework shows that for all the main elements, except ‘evaluation efforts,’ most of the participants stated that they have at least one of the sub-elements currently in place to support evidence-informed decision-making (Table 2).

**Table 2:** High level coding of the seven main elements currently in place to support evidence-informed decision-making

**Supports to facilitate evidence-informed decision-making**	**Number of interview participants (n)**^**1**^								
	**Total (n = 57)**	**Organization**			**Province**		**Occupation**		
		**PCP (n = 9)**	**Hospital (n = 32)**	**RHA (n = 16)**	**Ontario (n = 29)**	**Quebec (n = 28)**	**Senior management (n = 33)**	**Knowledge broker (n = 11)**	**Library manager (n = 13)**
Climate for research use	**57**	**9**	**32**	**16**	**29**	**28**	**33**	**11**	**13**
Research production	***52***	**9**	***30***	***13***	***27***	***25***	***31***	***9***	***12***
Push efforts	***52***	**9**	***31***	***12***	***24***	**28**	***31***	***8***	**13**
Facilitating pull efforts	***55***	**9**	**32**	***14***	***28***	***27***	***31***	**11**	**13**
Pull efforts	***56***	**9**	***31***	**16**	**29**	***27***	**33**	***10***	**13**
Linkage and exchange efforts	***55***	***8***	***31***	**16**	***28***	***27***	***31***	**11**	**13**
Evaluation efforts to link research to action	***39***	*4*	***22***	***13***	***21***	*18*	***24***	*7*	*8*

^1^Table legend. Percentage of total number of participants (font style used): 100% of participants (bold); 67% to 99% of participants (bold and italic); 33% to 66% of participants (regular i.e. not bold and not italic); 1% to 32% of participants (italic); 0% of participants (regular and underlined).

### Supports currently in place in healthcare organizations to facilitate evidence-informed decision-making

#### Establishing a climate for research use

Most participants highlighted the importance of “developing and implementing an infrastructure or positions where the accountability for encouraging knowledge use lies,” making this the most common element in all organization types (Table 3). Having specific positions in place that can support the use of evidence was viewed as essential: “the expertise in the resources we have available to us - that we have an epidemiologist and decision support individual who are capable of taking data and converting it to information.” Some hospitals even “got together; put some money together to support a support decision person to lead our decision support collaborative.” Infrastructures or positions that were mentioned by participants included a library or documentation centre and a department focused on KT (*i.e*., health technology assessment or a quality assurance department). Ontario participants noted that the rise in the establishment of quality assurance departments was most likely because of the provincial government’s increased focus on quality: “As far as the quality office, that is fairly new and I think was driven […] by the province’s move to implementation to quality improvement plans in hospitals in this past year.” A difference in response proportions was found for the “existence of a documentation or library centre” element, between knowledge brokers (45%) and library managers (100%).

**Table 3:** Establishing a climate for research use

**Supports to facilitate evidence-informed decision-making**	**Number of interview participants (n)**^**3**^								
		**Organization**			**Province**		**Occupation**		
	**Total (n = 57)**	**PCP (n = 9)**	**Hospital (n = 32)**	**RHA (n = 16)**	**Ontario (n = 29)**	**Quebec (n = 28)**	**Senior management (n = 33)**	**Knowledge broker (n = 11)**	**Library manager (n = 13)**
Develop and implement an infrastructure or positions where the accountability for encouraging knowledge use lies^1^	***55***	***8***	**32**	***15***	***27***	**28**	***32***	***10***	**13**
Emphasize the value of research use in decision-making in the mission, vision, values, and strategic plan	***50***	5	***31***	***14***	**29**	***21***	***31***	***10***	***9***
Build awareness of clear points of contact within the organization regarding where to turn to in order to acquire, assess, adapt and apply research evidence in decision-making processes	***38***	5	***23***	10	***21***	17	21	7	***10***
Place value on accreditation components that address the use of research evidence in decision-making^2^	29	3	***22***	*4*	18	11	21	4	*4*
Recognize in recruitment and retention strategies the value of having staff that have the ability or the desire to acquire, access, adapt, and apply research evidence in the decision-making process	27	3	15	9	15	12	18	5	*4*
Promote staff development opportunities that enhance skills in acquiring, assessing, adapting and applying evidence in decision-making	22	3	17	*2*	16	*6*	14	*3*	5
Organizations implement recognition programs that acknowledge and reward staff who use research evidence in decision-making	*17*	3	11	*3*	*7*	10	*10*	4	*3*
Integrate in performance reviews and appraisals key skills in acquiring, assessing, adapting and applying evidence in decision-making	*12*	*2*	*8*	*2*	*8*	*4*	*9*	*2*	*1*

^1^Different infrastructures mentioned in this node were: library or documentation centre (n = 36), department or section dedicated to KT (n = 24), and department for quality assurance (n = 15).

^2^A recurring theme mentioned in this node was ‘organization tends to encourage KT because use of evidence is evaluated in accreditation process’ (n = 14).

^3^Table legend. Percentage of total number of participants (font style used): 100% of participants (bold); 67% to 99% of participants (bold and italic); 33% to 66% of participants (regular i.e. not bold and not italic); 1% to 32% of participants (italic); 0% of participants (regular and underlined).

Most participants also highlighted that their “organization emphasizes the value of research use in decision-making in the mission, vision, values, and strategic plan”: “the language of the strategic plan indicates that we will be following evidence-based practice and that requires that you do research and your background work on your goals and objectives within the organization.”

Most participants also stated that their organization “builds awareness of clear points of contact within the organization regarding where to turn to in order to acquire, assess, adapt and apply research evidence in decision-making processes.” Clear points of contact that were most frequently mentioned by participants were librarians; however, epidemiologists and data specialists were also mentioned. The librarians were viewed as integral figures in facilitating the use of research evidence; “we have a marvelous librarian and she is very open …and she’s very prompt and so I think we’re very lucky to have that kind of position, particularly in a community hospital.”

#### Producing and participating in research

Many participants stated that their “organization participates in the production of primary research, reviews and research-derived products” (Table 4). For example; “we had an ambulatory redesign project, where senior management was committed to redesigning our ambulatory care program, and they engaged our group […] of researchers to say “if we're going to do this let's do it with some rigor. So they initiated that and then are very interested in taking the results of that research and feeding it back into the organization, both in terms of improving the patient experience, but also looking at how and if we should be supporting diverse platforms of patient engagement*.*” A difference in responses was found for this element between participants from PCPs (89%) and those from RHAs (38%).

**Table 4:** Research production

**Supports to facilitate evidence-informed decision-making**		**Number of interview participants (n)**^**2**^							
	**Total (n = 57)**	**Organization**			**Province**		**Occupation**		
		**PCP (n = 9)**	**Hospital (n = 32)**	**RHA (n = 16)**	**Ontario (n = 29)**	**Quebec (n = 28)**	**Senior management (n = 33)**	**Knowledge broker (n = 11)**	**Library manager (n = 13)**
Participate in the production of primary research, reviews and research-derived products	37	***8***	***23***	6	17	***20***	19	6	***12***
Partner as decision-makers on research or provide matching funding for priority projects^1^	37	***8***	21	8	***21***	16	***25***	***8***	*4*
Participate in regular priority-setting processes to ensure research meets needs of decision-makers	25	*1*	17	7	15	10	18	4	*3*
Ensure research commissioning capacity exists to commission or execute high priority research	25	***6***	15	*4*	13	12	18	4	*3*

^1^Three sub-elements that were mentioned were: establishing a research institute on site (n = 19), funding projects through partnerships (n = 16), and making available institutional funds for research (n = 14).

^2^Table legend. Percentage of total number of participants (font style used): 100% of participants (bold); 67% to 99% of participants (bold and italic); 33% to 66% of participants (regular i.e. not bold and not italic); 1% to 32% of participants (italic); 0% of participants (regular and underlined).

Many participants also stated that the organization’s willingness to partner as decision-makers on research or provide matching funding for priority projects facilitated the use of research in decision-making; “[Universities] rely on the hospitals a lot to help them with their teaching and graduate student supervision […] we decided that those projects would have to be […] projects we needed to have done. So we elicit[ed] ideas from our group and this year it even extended outside of nursing to infection control because there are nurses working there. And then we select […] the top priority projects in terms of the issues we need to have dealt with and how it fits with the department’s priorities and the hospital’s priorities and then our clinicians serve, if they are at least masters’ prepared, they serve as the supervisor for those students.”

#### Implementing ‘push’ efforts

Many participants stated that they had a “knowledge intelligence service that scans the literature and distributes research evidence throughout the organization” such as information monitoring services and electronic mailing lists to disseminate research results; “We have specific information monitoring set up for those who request it. We also have some staff involved in producing a general information bulletin that also looks to identify staff that may be interested by certain topics […] we see an article about their subject, we send it to that person or to the research team. We let them know we spotted the article and that it may be of interest to them.”

Many participants also stated that their organization “publishes and disseminates local research results,” notably through presentations within the organization and also to other organizations (Table 5).

**Table 5:** Implementing push efforts

**Support to facilitate evidence-informed decision-making**		**Number of interview participants (n)**^**3**^							
	**Total (n = 57)**	**Organization**			**Province**		**Occupation**		
		**PCP (n = 9)**	**Hospital (n = 32)**	**RHA (n = 16)**	**Ontario (n = 29)**	**Quebec (n = 28)**	**Senior management (n = 33)**	**Knowledge broker (n = 11)**	**Library manager (n = 13)**
Use a knowledge intelligence service that scans the literature and distributes research evidence throughout the organization^1^	36	***6***	***23***	7	15	***21***	19	6	***11***
Publish and disseminate local research results^2^	24	5	12	7	*6*	18	14	4	6
Ensure there are individuals responsible for identifying teaching moments to provide research evidence	*17*	0	14	*3*	*12*	*5*	*11*	*2*	*4*
Institute communications and marketing efforts related to research evidence	*16*	1	14	*1*	*6*	10	*6*	*2*	8

^1^Three sub-elements that were mentioned were: information monitoring services (n = 22), using a knowledge brokering service (n = 14), and electronic mailing lists to disseminate local research results (n = 13).

^2^Two sub-elements that were mentioned were: Providing research presentations both within and outside the organization (n = 15), and Implementing services to ensure the dissemination of research (n = 12).

^3^Table legend. Percentage of total number of participants (font style used): 100% of participants (bold); 67% to 99% of participants (bold and italic); 33% to 66% of participants (regular i.e. not bold and not italic); 1% to 32% of participants (italic); 0% of participants (regular and underlined).

#### Implementation of facilitating pull efforts

Most participants stated that essential supports for the use of research in decision-making include the implementation of a technical infrastructure to support research use, ensuring that no restrictions are placed on staff access to online resources that may contain relevant research evidence, and providing easy access to journals and scientific literature either through bulk purchasing of subscriptions or promoting open-access resources (Table 6). Many participants also stated that their organization provides an Intranet site or clear links to websites with relevant research evidence.

**Table 6:** Implementing facilitating pull efforts

**Support to facilitate evidence-informed decision-making**		**Number of interview participants (n)**^**4**^							
	**Total (n = 57)**	**Organization**			**Province**		**Occupation**		
		**PCP (n = 9)**	**Hospital (n = 32)**	**RHA (n = 16)**	**Ontario (n = 29)**	**Quebec (n = 28)**	**Senior management (n = 33)**	**Knowledge broker (n = 11)**	**Library manager (n = 13)**
Implement a technical infrastructure to support research and to ensure no restrictions are placed on staff's access to online resources that may contain relevant research evidence^1^	***46***	***8***	***29***	9	***25***	***21***	***26***	7	**13**
Provide easy access to journals and scientific literature either through bulk purchasing of subscriptions or promoting open-access resources^2^	***42***	***7***	***23***	***12***	***22***	***20***	***23***	***8***	***11***
Provide an Intranet site or clear links to websites that provide one-stop shopping for relevant research evidence	36	4	21	***11***	***22***	14	21	7	8
Implement accessible and efficient systems to support the use of research in decision-making^3^	27	5	15	7	15	12	18	*3*	6
Provide or participate in presentations/ online/ or face-to-face briefings about specific reviews or review-derived products	*18*	4	11	*3*	16	*2*	12	*2*	*4*

^1^A sub-element was noted here that participants had access through the university’s library resources (n = 11).

^2^Sub-elements that were noted here were: electronic based resources (n = 28), access through a network (n = 25), and databases (n = 22).

^3^A sub-element mentioned here was utilizing decision support tools such as templates for policy or procedure development (n = 12).

^4^Table legend. Percentage of total number of participants (font style used): 100% of participants (bold); 67% to 99% of participants (bold and italic); 33% to 66% of participants (regular i.e. not bold and not italic); 1% to 32% of participants (italic); 0% of participants (regular and underlined).

Those individuals that have easy access have become dependent and recognize its benefits: “We have easy, easy access to the [name of an academic hospital] library system. I mean, that’s like a little miracle. When I talk to people from other hospitals that don’t have that, I just keep thinking – I don’t know how I would function.” Being able to access resources from one’s office is a huge benefit for managers and decision-makers: “Having the capacity to do literature reviews from our office without having to take a day down at the library or negotiate lending privileges or library access issues which was always a pain in the butt. Now …everybody has access to everything. That is incredible. If you want to look for a questionnaire, or a research tool, it’s amazing.”

Some participants stated that they had limited access to research evidence (*e.g*., insufficient journal subscriptions, slow download speeds, locks placed on websites, etc.), and that these issues can be a great barrier to the use of research in decision-making. One participant summarized the lack of access: “having all these on-line resources. I think it’s really important because you may be trained on how to use evidence but if you don’t have access to it…? You go to the Extra Fellowship and you come back all pumped up and energized and thinking yeah, yeah I need to use evidence for everything from now on, right? […] Then you come here and there’s no access […] Game over.” Some participants stated that although they may have access restrictions through their organization, they find ways to go around it (*i.e*., through accessing the local university’s databases by using colleagues’ login details).

#### Implementing pull efforts

Most participants mentioned that their organization provides “training and continuing education that focused on finding and using research evidence in decision-making” (Table 7). Training programs that were mentioned by many participants were health leadership programs (*i.e*., EXTRA, Dorothy Wylie Health Care Leadership program, the Canadian college for health leaders, the Rotman leadership program, the RNAO fellowship), and training provided by the library staff. A difference in responses was found for the ‘training provided by the library staff’ element, between the library managers (100%), senior managers (15%), and knowledge brokers (27%). Participants commented that having staff participate in training, specifically EXTRA, ensures that the mind-set of EIDM is at the forefront of managers and decision-makers: ‘the fact that we have four EXTRA fellows within the organization really helps us to create the touch points to constantly keep the importance of evidence-informed practice at meetings and in our discussions.’

**Table 7:** Implementing pull efforts

**Support to facilitate evidence-informed decision-making**		**Number of interview participants (n)**^**4**^							
	**Total (n = 57)**	**Organization**			**Province**		**Occupation**		
		**PCP (n = 9)**	**Hospital (n = 32)**	**RHA (n = 16)**	**Ontario (n = 29)**	**Quebec (n = 28)**	**Senior management (n = 33)**	**Knowledge broker (n = 11)**	**Library manager (n = 13)**
Enable training and continuing education that focus on finding and using research evidence in decision-making^1^	***46***	***8***	***29***	9	***21***	***25***	***26***	7	**13**
Use dedicated staff to pull research into decision-making^2^	35	4	***23***	8	***21***	14	20	7	8
Ensure decision-making processes that promote the use of research in decision-making^3^	34	*2*	20	***12***	***23***	11	***24***	7	*3*
Summarize or conduct primary research via a rapid response unit	22	3	*10*	9	16	*6*	12	5	5

^1^Sub-elements noted here were: sending staff to external training programs (n = 21), providing training by in-house library staff (n = 21), and adapting training to specific specialties or clienteles (n = 14).

^2^Sub-elements noted here were: the use of experts, knowledge brokers, or opinion leaders (n = 22); and use of library staff (n = 19).

^3^One sub-element mentioned here was having committees or groups that use evidence to improve patient care (n = 15).

^4^Table legend. Percentage of total number of participants (font style used): 100% of participants (bold); 67% to 99% of participants (bold and italic); 33% to 66% of participants (regular i.e. not bold and not italic); 1% to 32% of participants (italic); 0% of participants (regular and underlined).

Many participants also highlighted the sub-element of the ‘use of dedicated staff to pull research into decision-making,’ and the majority of the time, this ‘dedicated staff’ person was a librarian. Organizations that had librarians that could pull the relevant research for a specific question recognized the importance of this. Librarians did note that the staff that attended EXTRA did not need assistance since they were well-trained in accessing research evidence; however, other staff, once they recognized the need for research to inform their decisions, would turn to the librarians or the knowledge brokers to gather the necessary research evidence to inform the decision. The staff recognized the importance of EIDM, but they did not have either the time or skills to search for the research themselves, and therefore having a staff member or unit that can search and summarize the relevant research was useful: ‘I don’t know if resistance was the right word but it seemed like there was almost, again, “we’re really busy, we don’t have time to run around looking for research or how to figure out how to deal with it” […] One of the ideas that we had initially was some training and rudimentary systematic review methods. […] They didn’t want any training […] But it was funny to get the reaction “No, we don’t want to learn to do that. Can you just go off and do it for us?”’

#### Instituting linkage and exchange efforts

Most participants stated that their “organizations had established formal and informal ties to researchers and brokers outside the organization who can assist in integrating evidence into the decision-making process” (Table 8). These ties could have taken different forms such as: being part of groups outside the institution, such as being part of regional, provincial or national networks; or having links to individual researchers, experts, or opinion leaders. One participant highlighted the importance of these linkages, *i.e*., “When the researcher from (affiliated university) came around, it had a really positive impact and some seeds were sown. […] She was involved in one project in particular […] This project had an impact on our practices […] She’s connected with other research groups from affiliated universities… she’s like our link to the university world.” One difference in responses was found for the “being part of groups outside the institution” element, between RHAs (88%) and PCPs (22%).

**Table 8:** Linkage and exchange efforts

**Support to facilitate evidence-informed decision-making**		**Number of interview participants (n)**^**2**^							
		**Organization**			**Province**		**Occupation**		
	**Total (n = 57)**	**PCP (n = 9)**	**Hospital (n = 32)**	**RHA (n = 16)**	**Ontario (n = 29)**	**Quebec (n = 28)**	**Senior management (n = 33)**	**Knowledge broker (n = 11)**	**Library manager (n = 13)**
Establish formal and informal ties to researchers and brokers outside the organization who can assist in integrating evidence into decision-making^1^	***47***	***6***	***26***	***15***	***25***	***22***	***28***	**11**	8
Hold regular meetings that highlight relevant research	36	***7***	21	8	19	17	***22***	6	8
Conduct interactive workshops that focus on the use of research in decision-making	26	***7***	14	*5*	13	13	15	6	5

^1^Sub-elements noted here were: being part of groups outside the institution such as regional, provincial, or national networks (n = 33) and link to individual researchers, experts, or opinion leaders (n = 29).

^2^Table legend. Percentage of total number of participants (font style used): 100% of participants (bold); 67% to 99% of participants (bold and italic); 33% to 66% of participants (regular i.e. not bold and not italic); 1% to 32% of participants (italic); 0% of participants (regular and underlined).

Many participants stated that their organizations had “regular meetings that highlight relevant research that was either conducted by the organization, or research that was produced outside the organization but is either of interest and not immediately relevant to the organization, or is specifically relevant to a current organizational change or implementation.” These meetings helped institute a culture in which research is viewed as important and significant, even if not immediately relevant. These meetings were held either weekly, bi-weekly or monthly, were voluntary, and could have been part of journal clubs, medical rounds, or monthly quality meetings. Meetings would take different forms and could be facilitated in a way to ensure that research was talked about and was at the forefront of peoples’ minds: “The journal club is changing because some of us want the whole group to evolve towards a more critical reading of articles. We noticed that anyone could read an article. They can all read articles but our group needs to learn about different research types and different statistical approaches. We are informal leaders but we are also peers… Having access to a bunch of articles is dandy but you have to take it further than that. You have to learn to be critical of these articles. I think the journal club allows us to learn to critique publications.”

#### Evaluating efforts to promote evidence-informed decision-making

For the ‘evaluation efforts’ domain, many participants stated that their organizations had somehow participated in evaluation activities in order to link research to action. However, participants stated that past evaluations of knowledge translation initiatives aimed at improving clinical procedures, primarily examining clinical outcomes and rarely examining the effect of evidence use on managerial decisions or evaluating the process of utilizing evidence. A difference in responses was found for the “evaluation efforts” element between RHAs (81%) and PCPs (33%).

### Top three supports for evidence-informed decision-making in healthcare organizations

In the second part of the interview, participants were asked, of all the elements they currently have in place, which they thought were the top three most important elements to support EIDM in healthcare organization. Of the seven domains in our framework, most participants highlighted the importance of establishing a “climate for research use,” while many highlighted the importance of implementing “facilitating pull” or “linkage and exchange efforts” (Table 9).

**Table 9:** Top three elements currently in place to support evidence-informed decision-making

**Support to facilitate evidence-informed decision-making**		**Number of interview participants (n)**^**4**^							
	**Total (n = 57)**	**Organization**			**Province**		**Occupation**		
		**PCP (n = 9)**	**Hospital (n = 32)**	**RHA (n = 16)**	**Ontario (n = 29)**	**Quebec (n = 28)**	**Senior management (n = 33)**	**Knowledge broker (n = 11)**	**Library manager (n = 13)**
**Climate for research use**	***39***	***7***	***23***	9	***23***	16	***23***	***9***	7
Organizations develop and implement a formal infrastructure or positions wherein the accountability for encouraging knowledge use lies^1^	27	5	18	*4*	14	13	13	7	7
Organizations emphasize the value of research use in decision-making in the mission, vision, values and strategic plan	*17*	*1*	11	*5*	14	*3*	13	*2*	*2*
**Research production**	19	*2*	13	*4*	*12*	*7*	*13*	*4*	*2*
Organizations participate in the production of primary research, reviews and research-derived products	*12*	*1*	*10*	*1*	*6*	*6*	*7*	*3*	*2*
**Push efforts**	23	*2*	15	6	10	13	12	4	7
Organizations institute a knowledge intelligence service that scans the literature and distributes research evidence throughout the organization^2^	*14*	*1*	*9*	*4*	*5*	*9*	*8*	*2*	*4*
**Facilitating pull efforts**	32	4	18	10	16	16	15	6	11
Organizations implement a technical infrastructure to support research and ensure no restrictions are placed on staff's access to resources that may contain relevant research evidence	*17*	3	*10*	*4*	11	*6*	*9*	3	5
Organizations provide easy access to journals and scientific literature either through bulk purchasing of subscriptions or promoting open-access resources	*16*	*1*	11	*4*	*5*	11	*4*	3	***9***
Organizations provide an Intranet site or clear links to websites that provide one- stop shopping for relevant research evidence	*12*	*2*	*7*	*3*	*7*	*5*	*4*	3	5
**Pull efforts**	26	0	18	8	17	*9*	14	6	6
Organizations encourage the use of dedicated staff to pull research into decision-making	*13*	0	*10*	*3*	10	*3*	*5*	4	*4*
Organizations encourage or provide training and continuing education that focus on finding and using research evidence in decision-making	*12*	0	*10*	*2*	*6*	*6*	*7*	*1*	*4*
**Linkage and exchange efforts**	26	4	11	11	13	13	17	6	3
Organizations establish formal and informal ties to researchers and brokers outside the organization who can assist in acquiring, assessing, adapting or applying research evidence in the decision-making process^3^	21	*2*	*10*	9	12	*9*	13	5	*3*
**Evaluation efforts to link research to action**	*11*	*1*	*7*	*3*	*9*	*2*	*7*	*2*	*2*

^1^The sub-element mentioned here often was the organization implementing a library or documentation centre .(n = 15).

^2^A sub-element mentioned here was organizations implementing information monitoring services (n = 13).

^3^One sub-element mentioned here was having employees that are part of groups outside the organization (n = 15) such as national, regional or provincial networks.

^4^Table legend. Percentage of total number of participants (font style used): 100% of participants (bold); 67% to 99% of participants (bold and italic); 33% to 66% of participants (regular i.e. not bold and not italic); 1% to 32% of participants (italic); 0% of participants (regular and underlined).

Of the sub-elements, there were four that were viewed as the most important elements that are currently in place to support EIDM and they were:

1. Organizations develop and implement a formal infrastructure or positions wherein the accountability for encouraging knowledge use lies (a sub-element of ‘climate for research use’);

2. Organizations establish formal and informal (or strong and weak) ties to researchers and brokers outside the organization who can assist in acquiring, assessing, adapting or applying research evidence in the decision-making process (a sub-element of ‘linkage and exchange efforts’);

3. Organizations emphasize the value of research use in decision-making in the organization's mission, vision, values and strategic plan (a sub-element of ‘climate for research use’); and

4. Organizations implement a technical infrastructure to support research and ensure no restrictions are placed on staff’s access to online resources that may contain relevant research evidence (a sub-element of ‘facilitating pull efforts’).

A difference in responses was found for the “organizations provide easy access to journals and scientific literature either through bulk purchasing of subscriptions or promoting open-access resources,” between library managers (69%) and senior managers (12%).

Participants recognized that within all the elements mentioned, there needs to be some alignment and explicit effort to capture synergies between various components of the framework in order for there to be real use of research evidence in decision-making. Investing in one component of the framework will not enable real change. For example: ‘[What] I would say is that in a parallel fashion there has to be investment in the infrastructure to support the decision-making. So, the culture change and training are two pieces that are important but if you do those things and don’t put in the infrastructure to support the decision-making, so, you don’t build data systems that provide meaningful information to the decision-makers, then you’re just teaching them something in abstract that’s completely irrelevant.’ Also, ‘That interdependency is extremely important […] because unless the culture shifts a little bit at least you won’t even get their attention. Unless you educate them, I wouldn’t call it training, but say education a little bit they won’t make the investment […] This year we’re spending more on our IS, information infrastructure than we are on medical equipment.’

## Discussion

### Summary of study findings

In this study, we investigated the supports that three types of health system organizations (*i.e*., RHAs, hospitals and PCPs) in two Canadian provinces (*i.e*., Ontario and Quebec) currently have in place to facilitate EIDM. Based on thematic analysis of the data obtained from 57 interviews in 25 organizations, we found four main factors related to use of research in decision making.

1. “The organizational climate” was identified as one of the most important elements that could impact the use of research in decision-making, and within this element, developing and implementing an infrastructure or positions for encouraging knowledge use was identified as the most important. It was evident that participants recognized the value and importance of EIDM; however, they did not have either the time or skills to search for the research themselves, and therefore having a staff member or unit that can search and summarize the relevant research was useful.

2. ”The linkage and exchange efforts” within and across organizations and networks was highlighted as essential since it facilitated ease of access to necessary research, enhanced dialogues between researchers and users, and assisted in the establishment of a culture that valued research evidence, even if the research was not immediately relevant. It essentially provided a network of contacts and experts that could be accessed to obtain relevant research to incorporate into the decision-making process.

3. “The facilitating pull efforts,” most specifically, a technical infrastructure and the ability to access research evidence when and where it is needed, was also identified as an essential element that, if properly in place (i.e., limited access restrictions), can facilitate the use of research in decision-making.

4. “The pull efforts,” and more specifically, providing or enabling staff to participate in training programs, ensured that there were individuals within the organization who valued the use of research evidence in the decision-making process and also had the skills to acquire, assess, adapt and apply the evidence. This also ultimately fed into the organizational culture and the value of incorporating research into decision-making.

The findings of this study suggest that organizational commitments, coupled with the necessary infrastructures, tools and expertise, are essential supports needed to move healthcare organizations toward EIDM. These organizational efforts have to be sustained and evaluated to ensure that the supports align with decision-makers’ needs for evidence at the management level.

### Summary of differences in responses

Interviewing multiple respondents and positions within various healthcare organizations enabled the cross-validation of the data. As was demonstrated in the results section and in the Tables, the majority of the coded responses were in alignment, and the data between Ontario and Quebec is fairly aligned. There were limited large differences in responses, and when there were differences of greater than 50%, it was understandable due to different position types (lending to different viewpoints or exposure in the organizational hierarchy) or organization types (lending to different purposes and perspectives). For example, a difference was found for the ‘training provided by the library staff’ element, between the library managers (100%), senior managers (15%), and knowledge brokers (27%). This could be because, although it is available and offered by librarians, maybe it is not visible or properly marketed to senior management, and therefore different position types may have different views. Difference in organization types may also lead to differences in responses; for example, there was one difference between PCPs (22%) and RHAs (88%) with respect to ‘being part of a regional, provincial or national network*.*’ We hypothesize that this is most probably because RHAs operate on a regional/provincial level and exchange ideas and programs with different RHAs, whereas PCPs operate at a much more local/hospital level and would not be part of broader networks.

### Relation to other studies

To our knowledge, this is the first qualitative study with a comprehensive framework of possible supports for EIDM. Studies do exist that examine either: one type of health service organization *i.e*., mental health services and laboratory units; a small number of interventions; or focus on decisions made at the clinical level and not at the management level [19-23]. In our scoping review, which served as the background for the guiding framework for this research, we did not identify any studies discussing the effects of a full research knowledge infrastructure on the use of evidence by managers and policy-makers, but we did uncover 25 qualitative studies and one randomized control trial that addressed different components of a potential research knowledge infrastructure [14]. Studies, like the 26 that we identified in the scoping review, continue to be published, such as a recent one from a member of our research team examining the availability of scientific journals, databases, and health library services in health ministries in Canada [24]. To our knowledge, this study is the first of its kind where: a number of health service organizations *i.e*., primary care practices, hospitals, and regional health authorities were examined; one to three key actors in each organization were interviewed to gain a broad perspective as well as to ensure alignment of responses; the respondents were asked about a wide range of interventions that could be undertaken either by the organization or by the health system in order to facilitate evidence-informed decision-making; and the focus was on management decision-making and not clinical decision-making.

The framework for this research was built upon a scoping review that reviewed the current literature to identify infrastructural initiatives that organizations have implemented to support the use of evidence in decision-making [14]. Our findings in this research mirror what was discovered in the scoping review, *i.e*., in the scoping review, we found that most studies focussed on ‘establishing a climate for research use,’ and in this research, all participants mentioned having at least one element within this domain in their organization, and most categorized at least one sub-element of climate within the top three most important elements. The scoping review also showed that: the next most addressed domains in the literature were efforts focusing on ‘facilitating pull,’ ‘linkage and exchange,’ and ‘pull activities,’ which was also highly commented on in this research; and the domain least reported on in the literature, ‘evaluation efforts,’ was also the least commented on in this research.

The responses received from the participants regarding the most essential elements to support the use of research evidence in decision-making were consistent with other literature. First, ‘establishing a climate for research use’ was classified as an integral foundation on which to ensure the use of research in decision-making. Implementing an infrastructure to support the use of EIDM is an exercise in organizational change, and research has demonstrated that a supportive climate and culture is an essential foundation element to support change in general, as well as specifically to supporting EIDM [25-27]. The second most essential element highlighted by the participants was ‘implementing facilitating pull efforts,’ which includes the implementation of a supportive technical infrastructure and access to research evidence, articles and databases. Other research and frameworks [4,28-30] have demonstrated that the technical infrastructure needs to be in place in order to facilitate the use of research in decision-making, *i.e*., “Strategic goals, critical appraisal skills and enthusiasm for EIDM are of limited use if organizations lack the infrastructure to acquire research evidence” [31] p.9. The third and fourth most essential elements (both mentioned by many participants), ‘implementing pull efforts’ and ‘linkage and exchange efforts,’ were also supported by the literature. ‘Pull efforts’ (*i.e*., engaging knowledge brokers or sending staff to training programs) were mentioned frequently in the literature as integral factors in building a framework for EIDM [4,12,31-34]. Linkage and exchange efforts’ have been mentioned in numerous frameworks [4,14,27,35,36]. Strong links between decision-makers and researchers can enhance the transfer of research into practice [37], and as can be seen in this research, participants use these links to build their knowledge base and tap into when necessary.

Furthermore, strong links between decision-makers and research producers can enhance the type of research being produced, *i.e*., make it more relevant and highly applicable to the needs of the users, and ensure that the research addresses high priority issues [38,39]. As is discussed in the “two communities theory,” researchers and decision-makers live in two different communities, with different values, reward systems, and languages [40]. This needs to be addressed by increasing the linkage and exchange between the two groups to achieve a shared understanding, which can influence the agenda setting, the type of research conducted, and the transfer of research into practice [32,41].

Findings of this study are supported and complemented by other bodies of literature that examine sustainable system changes. In order to build a strong EIDM infrastructure, it is crucial to assess the environment and include in the system the supports identified in the scoping review [14]. There is agreement between the organizational change literature and with the findings in this study that instituting a change is multi-layered, multi-faceted, and multi-challenging [25,31,42]. This study provides evidence that a supportive climate is essential; however, that alone cannot ensure EIDM. Tools need to be implemented so that EIDM is supported, encouraged and utilized every time. Without the infrastructure, instruments and tools, EIDM will be difficult to achieve, and it will not occur in a consistent and repeated manner.

### Strengths and limitations

There are two main strengths related to this study. First, we interviewed up to four participants in three different positions from each organization, which increased our confidence in the presented data, enabled us to cross-validate responses, and facilitated obtaining a global view of what elements were in place in the different participating organizations. Furthermore, there was limited variation in participants’ responses by position or organization type. Second, participants were from the three main types of organizations within the healthcare sector that are responsible for funding and delivering the bulk of healthcare services in Ontario and Quebec.

There are four main limitations to this study. First, some participants, notably library managers, were mostly involved with others at the clinical level and were not able to provide us with much information on evidence-based decision-making at the managerial level. However, by interviewing more than one informant from each organization, we were able to get a broad view of what infrastructures were in place and viewed as important for evidence-based decision-making at the managerial level. Second, most participants from the same organization were not interviewed at the same time. A focus group may have provided more consistent data, yet participants may have hesitated to speak openly in front of others. Focus groups were not utilized due to costs and scheduling concerns. Third, there was poor recruitment from the PCPs, which could be because of the lack of human resources allocated to KT-related duties, as is evidenced by the absence of library staff or resources on site and of knowledge broker-like personnel. Finally, while our sampling strategy was intended to be quite thorough, we do recognize that we were examining a best case scenario at a certain point in time. While we do anticipate that other organizations can learn from the high performers, no comparisons were undertaken with provinces or organizations that have not yet invested in knowledge translation initiatives.

### Future research

The present study is the second phase in a broader program of research: the first phase being an environmental scan and scoping review, this second phase, and then the third phase that will consist of a large cross-sectional web survey among all RHAs and hospitals in Ontario and Quebec. This survey will provide a more in-depth and broader picture of the different supports implemented to facilitate evidence-informed decision-making in Canadian healthcare organizations. This research may serve as a springboard to cross-organization and cross-system research to better understand how to match particular supports to different contexts. The ultimate purpose of this research program is to develop context-specific interventions and then properly evaluate them to determine which interventions can facilitate the transfer of research evidence into decision-making.

Implementation research that can identify barriers and facilitators of different interventions is essential. However, research on the KT processes and potential tools that can facilitate the uptake of research into decision-making is also needed [43,44]. One domain that was not strong in any of the participant organizations was the domain of evaluating KT efforts. A review of the current literature suggests that there are not many evaluations of KT interventions at the organizational level. Future research should examine KT interventions, infrastructural components, and tools to identify which elements are successful in which contexts.

### Implications

The uptake of innovation and change in health system organizations has traditionally been a challenging process [25,42]. The present study focused on organizations that have already demonstrated strategic structures and processes to support evidence-informed policy-making. It identified which elements these organizations currently have in place and which are held to be most important. What is clear from this research is that many infrastructural interventions exist and that organizations should benefit by building an infrastructure that not only encourages but also supports the use of research in decision-making. Those organizations that want to institute EIDM may want to explore some of the top four interventions identified by the respondents in this research and pursue those interventions to increase the prospects of the uptake of EIDM.

While some of the interventions mentioned by the participants can be quite costly and difficult to develop and implement, they may be easily transferable between organizations. The health system (*i.e*., hospitals, networks, provincial and federal governments) may benefit from exploring the idea of either encouraging resource and idea sharing, or investing in some of the larger up front significant investments in order to ensure widespread dissemination usage. For example, investing in a one-stop shopping website or free access to journal articles are initiatives that larger organizations can facilitate, yet smaller organizations can also reap the benefits. Such steps may help to improve the use of research evidence.

## Competing interests

The authors declare that they have no competing interests.

## Authors’ contributions

MEE coordinated the study, conducted the interviews in Ontario, analyzed the data, and drafted the manuscript. GL and GB conducted the interviews in Quebec, analyzed the data, and assisted in drafting the manuscript. JNL conceived and designed the study, oversaw the scientific direction, and helped to draft the manuscript. MO contributed to the conception and design. JMG contributed to the conception and design of the study. All authors read and approved the final manuscript.
